# Current Understanding of the Biomechanics of Ventricular Tissues in Heart Failure

**DOI:** 10.3390/bioengineering7010002

**Published:** 2019-12-20

**Authors:** Wenqiang Liu, Zhijie Wang

**Affiliations:** 1 School of Biomedical Engineering, Colorado State University, Fort Collins, CO 80523, USA; Wenqiang.Liu@colostate.edu; 2 Department of Mechanical Engineering, Colorado State University, Fort Collins, CO 80523, USA

**Keywords:** myocardium, stiffness, viscoelastic property, anisotropy, fibrosis

## Abstract

Heart failure is the leading cause of death worldwide, and the most common cause of heart failure is ventricular dysfunction. It is well known that the ventricles are anisotropic and viscoelastic tissues and their mechanical properties change in diseased states. The tissue mechanical behavior is an important determinant of the function of ventricles. The aim of this paper is to review the current understanding of the biomechanics of ventricular tissues as well as the clinical significance. We present the common methods of the mechanical measurement of ventricles, the known ventricular mechanical properties including the viscoelasticity of the tissue, the existing computational models, and the clinical relevance of the ventricular mechanical properties. Lastly, we suggest some future research directions to elucidate the roles of the ventricular biomechanics in the ventricular dysfunction to inspire new therapies for heart failure patients.

## 1. Introduction

Despite the advances in modern management, heart failure (HF) leads to high mortality and morbidity in the United States. More than 5 million Americans have HF, and around 550,000 new cases occur every year [1,2]. It is shown that the lifetime risk for developing HF at the age of 40 years old is around 20%, and the risk of HF increases with aging. As the number of elderly (≥65 years old) is expected to grow to 70.3 million in 2030, the prevalence of HF will continue to increase [1,3,4,5]. Economically, HF is the leading cause of hospitalization [2], with more than $33 billion in expenses annually in the United States. [1]; and in developed countries, the burden of HF is likely to keep increasing [4,6]. 

Ventricle dysfunction is the most common cause of heart failure, including left-sided HF with preserved ejection fraction (HFpEF) and reduced ejection fraction (HFrEF), as well as right-sided HF secondary to pulmonary hypertension and congenital heart disease (CHD) [7,8,9,10,11,12,13,14]. The malfunction of the myocardium in these diseases can occur in the left ventricle (LV), right ventricle (RV), or both ventricles (biventricular HF). It is known that the LV and RV have distinct embryological, geometrical, and structural properties [15,16,17], and the mechanism of RV failure is likely to be different than that of LV failure [18]. However, compared with LV failure, RV failure has been less understood, and it remains unclear if the two ventricles present similar mechanical behaviors or adaptations in the pathogenesis of ventricular dysfunction. 

It is generally accepted that the mechanical property of the myocardium is an important determinant of the ventricular function [19,20]. Indeed, changes in the ventricular mechanical properties during the HF progression have been reported in numerous studies for both LVs and RVs. The alteration of the extracellular environment can result in the dysfunctions of cardiac cells, and thus the overall organ function is impaired, which forms a vicious cycle in the maladaptive remodeling of the ventricle. Therefore, it is critical to unravel the roles of the tissue biomechanics in the ventricular dysfunction to inspire new therapies for HF patients. In this review, we will summarize the methodologies of the mechanical measurement of ventricle free walls, as well as the current understanding of ventricular mechanical properties including the tissue viscoelasticity, the existing computational models, and the clinical relevance of the biomechanical properties of ventricles. In particular, we have discussions on the right ventricle and the dynamic mechanical properties of the tissue—viscoelasticity, both of which have received less attention in the current research on cardiac biomechanics. Finally, future directions are suggested to advance the understanding of the biomechanical mechanisms of the heart failure in systemic and pulmonary circulations.

## 2. Characterization of the Mechanical Behavior of Ventricles

### 2.1. Ex Vivo Measurements 

**Figure 1:**
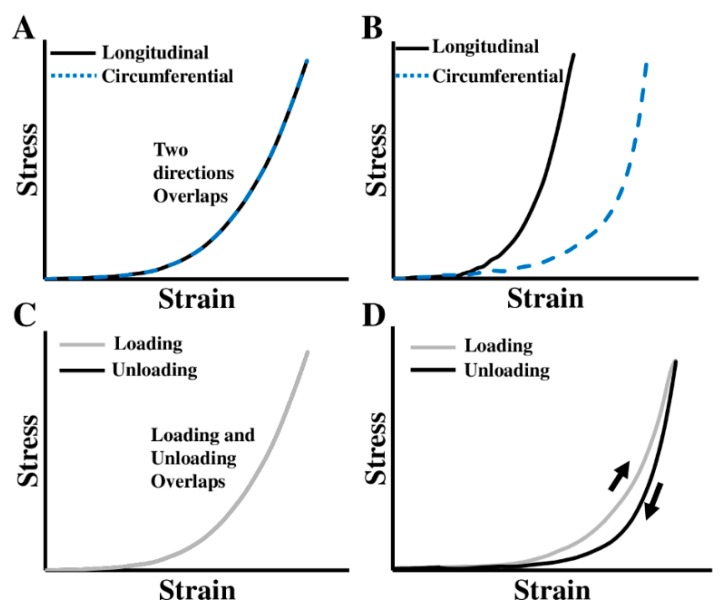
(**A**)–(**B**) Stress–strain curves obtained from different directions in isotropic and anisotropic materials, respectively; (**C**)–(**D**) stress–strain curves obtained from loading and unloading periods of cyclic deformation in nonlinear elastic and viscoelastic materials, respectively.

**Figure 2:**
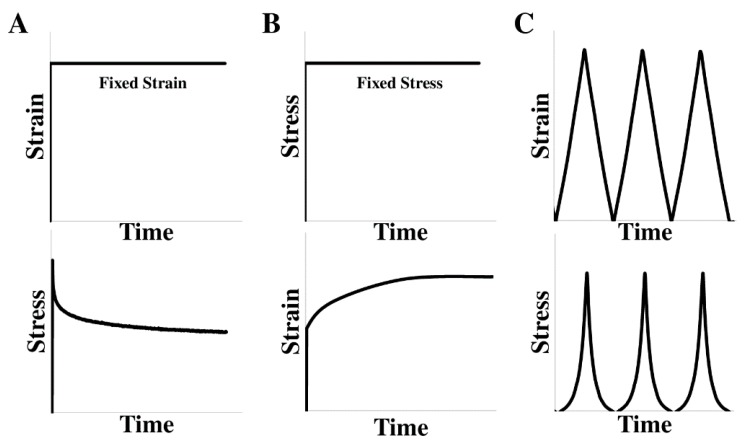
Different mechanical tests for the viscoelastic properties measurement. (**A**) Stress relaxation test, (**B**) creep test, and (**C**) displacement-controlled cyclic tensile mechanical test. The upper panels illustrate the mechanical inputs and the lower panels illustrate the mechanical responses of the material in these tests.

The ventricular free wall is known as an anisotropic and viscoelastic material, which means it has different mechanical behaviors in different directions and presents both elastic and viscous features in dynamic deformations (Figure 1). Depending on the mechanical behavior to measure, the mechanical tests can be uniaxial or biaxial (for anisotropic behavior), static or dynamic (for elastic or viscoelastic behavior), and in different testing conditions (e.g., bath medium, temperature, preconditioning protocol, removal of residue stress). To obtain the viscoelastic properties, either stress relaxation/creep tests or cyclic tensile mechanical tests can be used (Figure 2, detailed discussions in 2.1.2 and 2.1.3). Then, the viscous behavior is quantified to capture the time-, strain rate-, or frequency-dependent character [18]. In this review, we will focus on the macroscopic mechanical measurements, and thus the experimental methods using atomic force microscopy (AFM) or length-tension tests on the isolated cardiac muscle (e.g., papillary muscle) or cardiomyocytes are not included.

#### 2.1.1. Preconditioning and Residual Stress Measurement

As needed in other biological tissues’ mechanical tests, preconditioning is often performed prior to the data acquisition to ensure a constant and accurate mechanical behavior of the tissue [21]. This procedure has been described in the mechanical tests of cardiac tissues [17,22,23,24,25,26,27,28,29]. The number of preconditioning cycles in the biaxial/uniaxial tests varied among 5–10 cycles for the animal (canine, bovine, and murine) myocardium [17,22,24,25,26,27,28,29], whereas Sommer et al. and Fatemifar et al. showed that, after 3–5 cycles, the human heart tissue reached stable biaxial behavior [23,30]. Owing to the viscoelastic nature of the tissue, a sufficient resting period should be given between the tests. It is suggested that ten times of the previous mechanical testing period is appropriate for the tissue to be free from the ‘memory’ of former deformations [31,32]. 

Residual stress is the stress that remains in the tissue after all external loads are removed [33]. The presence of residual stress in myocardium has been observed in both large animal (porcine) and small animal (rat) ventricles [34,35,36,37]. The exact cause of residual stress in biological tissues is not fully clear, but the different growth rates at different layers or directions of the tissue are likely the reason [36]. Residual stress is generally considered ‘beneficial’ to the tissue. From the study of opening angle in an arterial ring, it is found that the presence of residual stress leads to a homogenous distribution of the circumferential wall stress through the vessel thickness [38]. For myocardium, Shi et al. measured the residual stress by a curling angle characterization and found that the residual stress protected the ventricle wall by reducing myocardial stress during LV diastolic expansion [34]. The measurement of residual stress in myocardium is seldom seen in ex vivo mechanical tests and future experimental studies may consider to include such measurement. 

#### 2.1.2. Uniaxial and Biaxial Tensile Mechanical Tests

**Figure 3:**
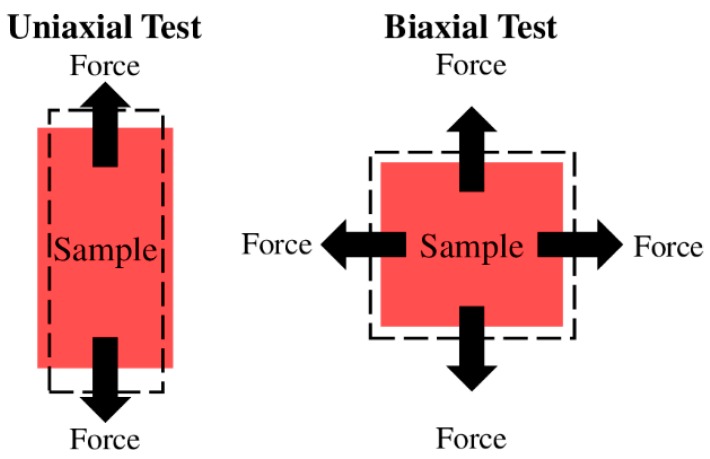
Diagrams of the uniaxial (**left**) and biaxial (**right**) tissue mechanical tests. Dashed rectangles illustrate the deformed configurations of the sample after the mechanical stretch.

Uniaxial and biaxial mechanical tests are the most common methods to investigate the ventricular mechanical property after tissue harvest (Figure 3). While the uniaxial mechanical test offers a quicker and easier examination of the material mechanical property, the biaxial mechanical test better mimics the in vivo loading conditions and provides more comprehensive measurements of the anisotropic mechanical behavior [17,19,20,22,39,40,41]. Both methods have been used in prior studies of LV and RV mechanical properties [17,20,22,23,24,25,26,27,28,29,30,39,40,41,42,43,44,45,46] (please see Table 1 for a summary of these studies).

**Figure 4:**
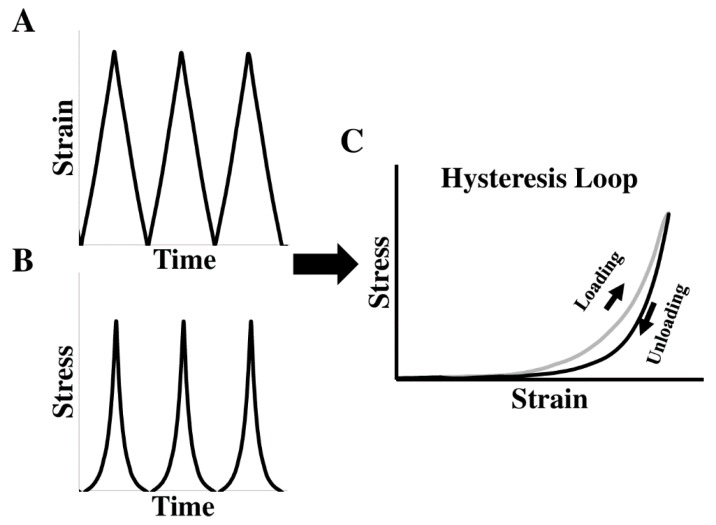
Hysteresis stress–strain loop obtained from the cyclic tensile mechanical tests. Triangle or sinusoidal mechanical loadings are typically applied during the cyclic stretches. (**A**)–(**B**) Representative strain and stress curves as a function of time in the tensile mechanical test; (**C**) representative hysteresis loop derived from the synchronized stresses and strains in (**A**) and (**B**).

Furthermore, when the entire cycle of stress–strain data is used (i.e., including loading and unloading curves), the ventricular viscoelastic behavior can be derived from the hysteresis stress–strain loop (Figure 4). However, the biaxial measurement of viscoelasticity is less common than the elasticity measurement and only sporadic studies have examined canine [39], porcine [46], and human ventricles [23]. Recently, the viscoelasticity of neonatal porcine LVs and RVs was obtained using the cyclic uniaxial mechanical tests. The myocardial hysteresis was quantified by the ratio of the area enclosed in the hysteresis loop over the area beneath the loading curve, but the elasticity of these ventricles was not quantified [46]. To our knowledge, the first human myocardium viscoelastic behavior quantified by biaxial testing was reported by Sommer et.al. [23]. Increased stress and hysteresis area were evident with increased stretch rate (from 3 mm/min to 30 mm/min), but no viscoelastic property (e.g., elasticity or viscosity) was quantified from these biaxial tests. 

#### 2.1.3. Stress Relaxation and Creep Tests

Stress relaxation and creep tests are traditional methods to measure the viscoelasticity of soft tissues such as tendon, cartilage, and heart valves [47,48,49,50,51,52,53]. The stress relaxation test is the recording of a time-dependent stress reduction under a fixed strain/stretch, whereas the creep test is the recording of a time-dependent strain increase under a constant stress/load (Figure 2) [18]. These methods have been applied to the myocardium or papillary muscle [23,54,55,56,57,58], although slightly different testing protocols and conditions were adopted (please see Table 2). 

### 2.2. In Vivo Measurements 

The ex vivo measurement discussed above can provide better controls of the experimental conditions (e.g., the strain rage, cardiac muscle tone) and eliminate the interference of physiological factors (e.g., blood pressure, heart rate, hormone levels) in the mechanical properties of the ventricle tissues. However, the ex vivo tests require tissue removal and are often limited by the contractile state of cardiomyocytes (passive only) and the configuration of the tissue (non-physiological stretches in the biaxial directions). Therefore, the in vivo measurements could provide useful information of the tissue mechanical behavior that is absent in the ex vivo conditions.

#### 2.2.1. The Elasticity Measurement

**Figure 5:**
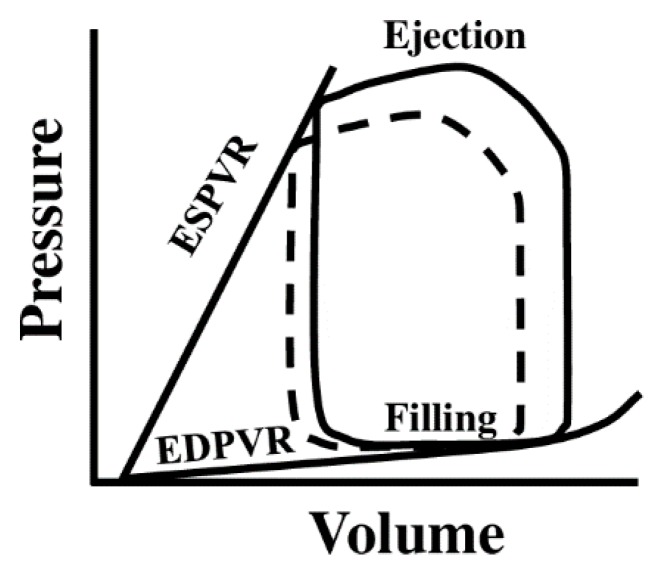
Diagram of the pressure–volume (PV) loop obtained from cardiac catheterization. The loop in solid line denotes a steady-state PV loop, whereas the loop in dotted line denotes a transient loop obtained by brief vena cava occlusion to reduce the ventricle filling. ESPVR: end-systolic pressure-volume relationship; EDPVR: end-diastolic pressure-volume relationship.

At the whole-organ level, pressure–volume (PV) loop measurement (Figure 5) is the gold standard to assess the ventricle performance invasively by inserting a PV catheter into the ventricle lumen [19]. The end-diastolic pressure–volume relation (EDPVR) derived from the steady-state PV loops is often used to represent the ventricular passive stiffness. Similarly, diastolic stiffness can also be estimated by the ratio of end-diastolic pressure (EDP) to end-diastolic volume (EDV) [59,60]. In addition, chamber compliance, which is the ratio of ventricular volume change over pressure change during a cardiac cycle (ΔV/ΔP), has also been used to describe the ventricle stiffness [61,62,63]. Another type of ventricular elastance, end-systolic pressure-volume relation (ESPVR or Ees), can be derived from a serious PV loops during a temporal vena cava occlusion or estimated by other formulas with a single beat technique. Ees is considered a measure of load-independent contractility of the ventricle [64]. However, this parameter has also been viewed as an index of *systolic* stiffness of the ventricle [59,60].

Non-invasively, cardiovascular magnetic resonance (CMR) and speckle-tracking echocardiography (STE) are alternative methods to measure the myocardial performance [65,66,67]. Depend on the imaging technique, 3D geometry is reconstructed and the strain and strain rate are then calculated as the indicators of ventricle stiffness. The in vivo 3D strain analyses can be achieved by applying a so-called hyperelastic warping method to various types of medical images such as cine CMR or echocardiography, from which global or regional myocardial strain can be calculated [68,69,70]. The hyperelastic warping method is a deformable image registration technique, which uses a deformable finite element mesh to register the target image to the reference image. The reference image is typically selected as the image at the end-diastole [71,72]. Then, the 3D deformation of the ventricular geometry can be derived over a cardiac cycle, and the strains in different directions (longitudinal, circumferential, and radial) are calculated [70,71,72,73]. This technique is powerful because it enables the measurement of the myocardial strain temporally and spatially, and both ventricles can be examined at the same time to further investigate the ventricular interactions in HF patients. These strain measurements could potentially offer new diagnostic or prognostic indices for LV or RV dysfunction [70,74]. However, it should be noted that the strain is essentially a measure of relevant deformation of the ventricular chamber, and such deformation is affected by both the passive stiffness and active contraction of the ventricular wall. Therefore, it is not a direct measurement of ventricular stiffness. 

The direct non-invasive measurement of ventricular stiffness (e.g., elastic modulus) can be obtained by magnetic resonance elastography (MRE) [75,76]. MRE is a phase contrast magnetic resonance imaging (MRI) technique. The underlying principle of this imaging method is based on the fact that the different stiffness of a material generates different shear wave length. With an induction of shear waves in the tissue region of interest, the waves are encoded in the phase of MR image and the wave images can be converted to the stiffness maps with temporal and spatial information included. MRE has been investigated in animals and a couple of clinical studies to study the effect of myocardial infarction, aging, hypertension, or hypertrophic cardiomyopathy on cardiac stiffness. A good review of MRE in cardiovascular tissues is given by Khan et. al [75]. However, although the methodology has been validated in animals with the gold standard PV loop, the elastic moduli reported in human subjects (<12 kPa) are much lower than the values reported in animals or in ex vivo measurements (in hundreds of kPa) [75]. Thus, more work is warranted in this area.

Finally, with the combination of medical imaging and computational modeling such as finite element methods, it is also possible to estimate the ventricular material properties using ‘inverse modeling’ [77,78,79,80,81,82]. These computational methods are briefly reviewed in the works of [83,84,85].

#### 2.2.2. The Viscoelasticity Measurement

The viscoelasticity of ventricles has been occasionally reported with the measurement of cyclic stress–strain relations. Some early studies measured the viscoelasticity of the LV from healthy canine and human hearts by individual measurements of pressure and volume in vivo [86,87,88]. Briefly, cardiac catheterization was performed and a micromanometer was introduced into the LV to measure the pressure. In the meanwhile, echocardiogram was performed and the endocardial diameter and the posterior wall thickness were recorded. These data were synchronized and further used to calculate the meridional wall stress and midwall strain during the diastolic phase. Viscoelastic properties in the ‘passive’ state of the LV were then derived from the nonlinear stress–strain curve using an empirical model of viscoelasticity. Interestingly, these studies were all published in the late 1970s and early 1980s, and there is no further investigation of the in vivo measurement of ventricular viscoelasticity.

### 2.3. Basic Behavior of Ventricles—Tissue with Anisotropy and Viscoelasticity

#### 2.3.1. Anisotropic Behavior of Ventricles

The characterization of the anisotropic behavior of ventricles is highly dependent on the definition of the biaxial coordinate system. To date, there are two main types of coordinate systems: the main fiber and cross-fiber coordinate system [17,30,44], and the outflow tract and cross-outflow tract coordinate system [20,29,40]. Using the former coordinate system, it is consistently observed that the tissue behaves stiffer in the fiber direction compared with the cross-fiber direction [22]. However, the degrees of anisotropy in the ventricles are not consistent among observations. Sacks et al. reported that the canine RV had greater anisotropy than the LV [17]. Similarly, Ahmad et al. found that the neonatal porcine RV had significantly greater anisotropy than the LV in different anatomic regions [89]. However, Javani et al. reported that the ovine LV was more anisotropic than the RV [44]. Ghaemi et al. reported that both LV and RV were anisotropic, but there was no comparison between these chambers [22]. Therefore, there is no consensus about the difference in anisotropic behavior between a healthy LV and RV. The discrepancies may depend on the age and species of samples, methods of tissue selection and preparation and testing protocols. Besides, it has been noted that the determination of the main fiber direction is challenging and could induce variations in the anisotropic behavior as well [17].

Using the second coordinate system, Valdez-Jasso et al. found that the rat RV had greater stiffness in the outflow tract direction compared with the cross-outflow tract direction [29], and Hill et al. found that the degree of rat RV anisotropy increased in the pressure overload state [40].

#### 2.3.2. Viscoelastic Behavior of Ventricles

**Figure 6:**
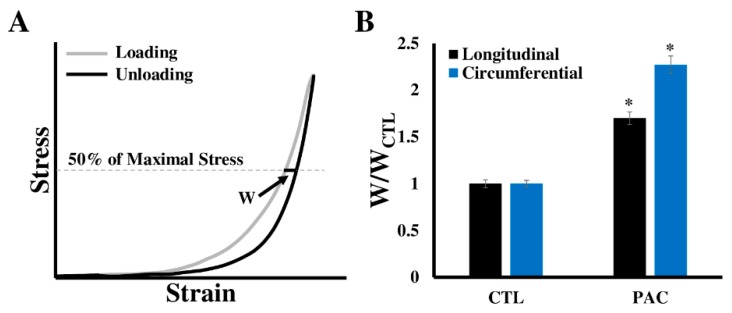
Change in the right ventricle (RV) viscosity after three-month pressure elevation in adult sheep. Pressure elevation was induced by pulmonary artery constriction (PAC). (**A**) Viscosity is defined as the loop width (w) at the 50% of the maximal stress of the loop; (**B**) Loop width normalized by the average loop width of the control RVs in the individual direction. CTL: control; PAC: pulmonary artery constriction. * *p* < 0.05 vs. CTL in the same direction.

The viscoelastic property of a material is manifested by the non-overlapping of loading and unloading stress–strain curves over an entire cycle [18]. Such behavior has been observed for both LV and RV tissues [18,39,90], which implies that the ventricular elasticity (or stiffness) is dependent on the strain rate, and there is energy loss during the cyclic deformation owing to the viscous property of the ventricle. Particularly, Ahmad et al. found that the neonatal porcine LV had greater viscoelasticity than the RV, and both ventricles exhibited greater viscoelasticity at the mean-fiber direction compared with the cross-fiber direction [46]. Sommer et al. measured the viscoelastic property of various diseased human LVs, and their findings also showed a larger hysteresis in the mean-fiber direction than the cross-fiber direction [23]. Our own recent study in ovine RVs showed that the chronic pressure overload increased hysteresis (viscosity) in both directions (unpublished data (Figure 6)). 

### 2.4. Computational Modeling of Ventricular Biomechanics

Both empirical models and constitutive models have been applied to characterize the nonlinear, biaxial mechanical behavior of ventricles. Because of the nonlinear, ‘J’-shaped stress–strain curve, the use of an exponential component is common in empirical models. However, these models provide little information on the relations of physical quantities or physiological conditions of the tissue, and thus constitutive models are developed to better describe the myocardium tissue mechanics [42]. With certain assumptions (hyperelasticity, incompressibility, homogeneity, and so on), a strain energy function is defined to relate the mechanical loadings (stress) to the geometry changes (strain). The determination of the strain energy function is the key in constitutive models. On the basis of the model parameters included in the strain energy function, different materials’ properties can then be derived. A thorough review of the modeling for tissues biaxial mechanical properties can be found in the works of [41,42].

Classic empirical models to describe the tissue viscoelasticity are composed of springs and dashpots that represent the elastic and viscous behaviors, respectively. The two basic models of these are also known as the Maxwell model (consisting of a spring and a dashpot in series) and Kelvin–Voight model (consisting of a spring and a dashpot arranged in parallel). Different combinations of the spring and dashpot elements have been used to describe the ventricle and papillary muscle viscoelasticity. For example, a spring connected to two Maxwell elements in parallel was used to form a 1D viscoelastic model for the papillary muscle of the LV [58]. An elastic term and a viscous term in parallel were used to describe the viscoelasticity of the LV in different conditions [39,87,88,91,92]. In the constitutive models of ventricular viscoelasticity, a finite element analysis with orthotropic viscoelastic model has been used to describe the passive myocardium viscoelastic behavior [93]. Another option to represent the viscoelastic behavior is by the hereditary (or convolution) integral with a strain-dependent Prony series, which has been found to successfully capture the strain- and time-dependent behavior in *non-cardiovascular* tissues [51,94,95,96]. A nice review of constitutive models of cardiac tissue viscoelasticity can be found in the literature [93,97,98].

## 3. Biomechanical Changes of Ventricles in Heart Failure Development

Heart failure is associated with extensive remodeling of the tissue involving changes in extracellular matrix (ECM) (e.g., fibrosis or accumulation of collagen), recruitment of inflammatory cells (e.g., macrophage infiltration), upregulated oxidative stress (e.g., increased ROS), and altered metabolic activity (e.g., increased glycolysis) [99,100,101,102]. These changes not only lead to the malfunction of various cells in the myocardium, but also result in the impairment in the mechanical and hemodynamic functions of the organ. Because of our focus on the biomechanical behavior of the ventricle in this review, we will restrict our discussions to the extracellular matrix (ECM) proteins (particularly collagen) as they are the main determinant of mechanical properties including viscoelasticity [18,92,103].

The myocardium ECM consists of proteins such as collagen, elastin, fibronectin, proteoglycan, and laminin. Among these molecules, collagen is the most abundant ECM protein in the adult heart, with at least five different types of collagen (I, III, IV, V, and VI) that have been identified [104]. Types IV and V collagen are mostly found in the basement membrane of the cardiomyocytes, and types I and III collagen are the main constituents in the ECM: type I collagen represents 75%~80% of total collagen content and type III collagen represents approximately 15%~20% of the total collagen [100]. The collagen metabolism, that is, the balance of collagen synthesis and degradation, is regulated by the mechanical loadings (i.e., pressure-overload, volume-overload) and leads to rapid changes in cardiac ECM and mechanical properties [105,106,107].

Ventricular fibrosis (i.e., collagen accumulation) is frequently observed in cardiac remodeling in both LV failure and RV failure [104,108,109], and the cessation of the accumulation or cross-linking of collagen has been shown to reverse the maladaptive remodeling and improve ventricular function [107,110,111]. However, the story about collagen accumulation is not as simple as firstly viewed if more aspects are considered. For example, in the late stage of HF with LV dilation and wall thinning, conflicting results are given in collagen metabolism: some report that (type I) collagen is degraded and the extent of collagen cross-linking is reduced [106,112,113], whereas other report elevated collagen content or cross-linking [111,114]. In response to pressure overload, the findings on LV collagen deposition are not consistent either: increased collagen [115], decreased collagen [116,117], and no change in collagen [118] in the ventricles were all reported. During the progression of RV dysfunction in pulmonary hypertension, the total collagen was increased with respect to time, but the percentage of collagen cross-linking was decreased [61]. This suggests that the role of collagen content and cross-linking in RV dysfunction may be different. Overall, the variations in collagen deposition depending on the etiology or the specific phase of the heart disease development suggest that collagen metabolism is a key factor contributing to the heterogeneity of the heart failure. Therefore, further examination of the collagen metabolism in LV/RV failure progression is required.

**Figure 7:**
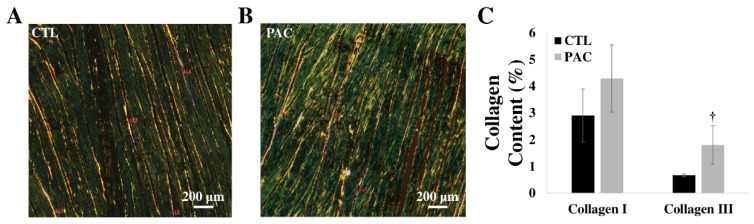
Collagen accumulation in hypertensive ovine RVs. (**A**)–(**B**) Representative histology images of the Picro Sirius Red staining of RVs in control and hypertensive groups, respectively; (**C**) Increase in type III collagen in the hypertensive RVs. CTL: control; PAC: pulmonary artery constriction. † p = 0.05 vs. CTL.

While these previous studies investigated the role of fibrosis in the HF progression, the link of collagen deposition to the mechanical changes is another open area of research. Some biomechanical studies have quantified both biaxial mechanical properties and collagen/myo-fiber orientation in the ventricle (mouse RV, infarcted LV) [23,29,45]. However, how the collagen orientation or total amount is correlated with the ventricular anisotropy or elasticity remains unknown. We recently exposed the ovine RVs to pressure overload using a pulmonary artery constriction model. The chronic remodeling of the RV led to increased collagen deposition. More interestingly, we observed a larger increase in type III collagen than in type I collagen (unpublished data) (Figure 7). Further investigations on the structure–function relations of the ventricles in different physiological conditions will provide more insights into the role of fibrosis in heart failure development. 

## 4. Clinical Relevance of Ventricular Mechanical Alterations

### 4.1. Significance of Ventricular Stiffening in Heart Failure

In chronic heart diseases, the myocardial structure and morphology changes lead to the stiffening of the ventricles [7,8,9,119,120,121]. These mechanical changes are considered as the changes in the passive mechanical behavior of the tissue, which is often related to the diastolic dysfunction [87,122]. The stiffening of the ventricle impedes the filling of blood during diastole, and thus leads to an increased filling pressure (EDP) at the same chamber volume. This is a key mechanism for the progression of LV dysfunction, particularly in heart failure with preserved ejection fraction (HFpEF) [122,123]. Recently, it was further demonstrated that the increase in passive stiffness proceeds the LV diastolic dysfunction [124]. Consequently, reducing LV stiffness has become one therapeutic target for HFpEF patients [125]. Ventricular stiffening also occurs in other conditions such as hypertension, aging, and hypertrophic cardiomyopathy [59,75], and the former two conditions are well-known risk factors of heart failure. 

In addition, the increased passive stiffness could result in an increase in stiffness during the systolic contraction, which is why the age-related increases in Ees (ESPVR, the elastance at systole) and EDPVR are correlated, regardless of the changes in arterial load [60]. While Ees is considered as a measure of ventricular contractility, it is possible that the systolic function of the ventricle is affected by the passive stiffness. Indeed, reduced LV strains in the longitudinal and circumferential directions have been reported in HFpEF patients compared with normal and hypertensive heart disease patients, which indicates the stiffening of the LV. Furthermore, these strains were correlated to the LV systolic function (ejection fraction), but not the diastolic function (E’ or E/E’), suggesting a link of the LV strain (indicator of stiffness) with the systolic performance [126]. However, whether and how the systolic function is altered by the increased passive stiffness in different etiologies of LV failure remains largely unexplored. 

Finally, the stiffening of LV could impact on the pulmonary circulation as well. Pulmonary edema and elevation in pulmonary venous pressures are observed as a result of the backward transmission of elevated left-sided pressures into the pulmonary circulation. This leads to the development of post-capillary pulmonary hypertension (PH), which is commonly found in HFpEF patients [127]. Therefore, both ventricles become dysfunctional, and this is probably why HFpEF is a more challenging type of heart failure to manage.

RV stiffening is consistently observed in a variety of PH etiologies as well as left-sided heart failure. Using non-invasive echocardiography, reduced RV longitudinal strains have been reported in pre-capillary PH (pulmonary arterial hypertension) patients and PH patients with other etiologies [128,129,130]. Increased RV stiffness was frequently reported in the preclinical studies of PH via the ex vivo tissue mechanical tests [20,29,40]. However, the impact of RV stiffening in the ventricular performance is rarely investigated. Recently, a correlation of RV longitudinal elastic modulus and the end-diastolic volume (EDV) was found in rodent RVs during PH development [20]. This is the first study to correlate the RV mechanics to the hemodynamic function of the organ. In another study of patient-specific biventricular constitutive modeling, a ratio of RVEDV/LVEDV was found to increase with increased RV free wall stiffness in PH patients, and this new index was strongly and inversely correlated with the RV peak contractility [131]. A following study from the same group suggested that this index can be used to estimate RV contractility [74]. Therefore, in both the left and right sides of the heart, the passive mechanical behavior is linked to the diastolic function as well as the contractility of the ventricle. This suggests that the improvement in the tissue mechanics may be a therapeutic target for heart failure patients.

### 4.2. Significance of Altered Ventricular Viscoelasticity in Heart Failure

The viscoelastic properties of the ventricle can impact the in vivo function. To date, the discussion of the relevance of ventricular viscoelasticity is mainly restricted to the diastolic function. Firstly, because the viscoelastic property is strain-rate dependent and because the early and later diastole have different filling rates, the diastolic function of the ventricle is time-dependent [86,87,132,133]. Furthermore, evidence has shown that the viscoelasticity of the ventricle changes from normal to diseased states. Increased viscosity of the LV has been reported in different types of patients (severe aortic regurgitation, congestive cardiomyopathy with preserved and reduced ejection fraction) with dilated, hypertrophy LVs [86]. Our preliminary data in pressure-overloaded ovine RVs also showed an increased viscosity in both biaxial directions compared with the healthy RVs (Figure 6). While these data indicate a change of tissue viscoelasticity in HF progression, the exact role of the viscous property in the ventricular function is not well understood.

## 5. Future Directions

It is well accepted that the passive mechanical properties of the ventricle are important for the diastolic function, and thus heart diseases with a change in myocardial mechanical properties are often associated with diastolic dysfunction [134,135,136,137,138]. However, if and how much of the systolic function is affected by the passive mechanical properties remain unclear. Second, the energy consumption of the tissue could also be affected by the mechanical properties of the tissue (e.g., viscosity) as the cyclic deformation involves energy storage, release, and dissipation. It is thus necessary to explore the energy consumption at the tissue level and how the use of energy at the organ/tissue level is related to the metabolism of individual cardiomyocytes. Overall, the comprehensive understanding of the relationship between the mechanical behavior and the ventricle performance awaits further investigations. Third, the research on ventricular viscoelasticity has been limited in the current literature. Future studies should characterize the viscoelastic properties of the ventricles at different physiological and pathological conditions and elucidate the role of acellular and cellular components in tissue viscoelastic properties. Finally, the RV, known as the ‘forgotten chamber’, has been less investigated compared with the left compartment. The understanding of the mechanical properties of the RV and their changes in RV failure progression will deepen the insights of the pathogenesis of RV failure or biventricular failure.

## Figures and Tables

**Table 1:** Summary of the prior biaxial/uniaxial tensile mechanical tests performed in ventricular tissues. The experimental details on testing methods and conditions are listed as well. Viscoelastic mechanical studies are marked with * in the Methods. CPS: Cardioplegia Solution; BDM: 2,3-butanedione monoxime; PBS: Phosphate-buffered saline.

Sample	Method	Axial Definition	Preconditioning Cycles	Strain Range/Rate	Bath Medium	Temperature	Immerse Condition
*Canine RV* [17]	Biaxial	Main fiber direction	10	30% /	Water with recycle required oxygenated carioplegic solution	Room temperature	Immersed
*Rat RV* [20]	Biaxial	Outflow tract	/	/ /	Modified Kreb’s solution with 2,3-butanedione 2-monoxime and oxygen	/	Immersed
*Bovine LV/RV* [22]	Biaxial and uniaxial	Main fiber direction	5	20% 0.10.75 cm/s	Saline with O_2_ and CO_2_ (pH = 7.4)	Physiological range	Immersed
*Human LV/RV* [23]	Biaxial* and Triaxial	Main fiber direction	4	20% Quasi-static	CPS with 20 mM BDM	37 °C	Immersed
*Canine LV* [24]	Biaxial	Main fiber direction	≥7	/ 50s/cycle	Modified Kreb’s Ringers solution with a ~ 10 mM potassium, O_2_, and CO_2_ (pH = 7.4)	30 °C	Float
*Canine LV* [25]	Biaxial	Main fiber direction	5–7	20% 0.05 or 0.1Hz	Bath containing the oxygenated solution	Room temperature	Immersed
*Canine LV* [26]	Biaxial	Main fiber direction	7–10	5%–27% 0.1 Hz	Oxygenated cardioplegic solution	Room temperature	Immersed
*Rabbit LV* [27]	Biaxial	Main fiber direction	Several	/	BDM–Krebs solution	/	Immersed
*Ovine LV* [28]	Biaxial	/	10	20%–25% 0.5 Hz	Isotonic cardioplegic solution (pH:7.4)	20 °C	Immersed
*Murine RV* [29]	Biaxial	Outflow tract	10	5–25 kPa /	Modified Kreb’s solution with BDM	Room temperature	Immersed
*Human LV/RV* [30]	Biaxial and uniaxial	Main fiber direction	5	40% ~6 mm/min	Phosphate-buffered saline (PBS)	37 °C	Immersed
*Canine LV* [39]	Biaxial and uniaxial*	Main fiber direction	9	/ 0.0025–0.25 mm/s	Tyrode solution with O_2_ and CO_2_ (pH:7.4)	29.5–30.5 °C	Float
*Rat RV* [40]	Biaxial	Outflow tract	/	/ /	Modified Kreb’s solution with BDM and oxygen	Room temperature	Immersed
*Canine LV/RV* [43]	Biaxial	Apex to base	/	/ /	Oxygenated solution	Room temperature	Immersed
*Ovine LV/RV* [44]	Biaxial	Main fiber direction	10	40% 8 s per cycle	Saline bath	37 °C	Immersed
*Rat LV* [45]	Biaxial and uniaxial	/	10	/ 0.5mm/s	PBS	37 °C	Submerged
*Porcine LV/RV* [46]	Biaxial and uniaxial*	Main fiber direction	/	/ 0.5mm/s	PBS	37 °C	Submerged

**Table 2:** Summary of the prior studies with stress relaxation or creep tests on ventricular tissues. LV, left ventricle; RV, right ventricle. CPS: Cardioplegia Solution; BDM: 2,3-butanedione monoxime; PBS: Phosphate-buffered saline; KHB: Krebs-Henseleit buffer.

Sample	Method	Ramp Speed	Stretch Level	Duration	Bath Condition
*Human LV/RV* [23]	Stress relaxation	100 mm/min	10%	5 min	CPS with 20 mM BDM at 37 °C
*Rabbit LV papillary muscle* [54]	Stress relaxation and creep	/	/	5 min	Ringer–Lacke solution with O_2_, CO_2_, pH = 7.38
*Cats, Rabbits papillary muscle; Frog and Turtle LV* [55]	Stress relaxation and creep	/	20%, 30%	/	Tyrode solution with O_2_, CO_2_, pH = 7.3, at 24 °C (for papillary muscles); Modified PBS solution at pH = 7.3 (for LVs)
*Chicken embryonic heart* [56]	Stress relaxation	Fast linear	10%, 20%, 40%	10 min	Oxygenated KHB–CPS at 35 °C
*Chicken LV/RV* [57]	Stress relaxation	1000% axial strain/s	5%, 10%, 20%, 30%	5 min	Oxygenated KHB–CPS at 35 °C
*Cat LV papillary muscle* [58]	Stress relaxation	/	/	/	Oxygenated Kreb’s–Ringer’s solution at 20 °C
